# Gender differences in hypoglycaemia in type 1 and insulin-treated type 2 diabetes: the Hypo-METRICS study

**DOI:** 10.1007/s00125-026-06775-6

**Published:** 2026-06-20

**Authors:** Aisling McCarthy, Gráinne Kent, Jonah Thomas, Gilberte Martine-Edith, Natalie Zaremba, Eduardo Avila, Stephanie A. Amiel, François Pouwer, Julia K. Mader, Patrick Divilly, Pratik Choudhary

**Affiliations:** 1 Department of Medicine, University College Dublin, Dublin, Ireland; 2 School of Population Health, Royal College of Surgeons, Dublin, Ireland; 3 Diabetes Research Centre, University of Leicester, Leicester, UK; 4 Department of Diabetes, School of Cardiovascular and Metabolic Medicine and Sciences, Faculty of Life Sciences and Medicine, King’s College London, London, UK; 5 Department of Medical Psychology, Radboud University Medical Centre, Nijmegen, the Netherlands; 6 Department of Computer Sciences, Maynooth University, Kildare, Ireland; 7 Department of Psychology, University of Southern Denmark, Odense, Denmark; 8 Steno Diabetes Centre Odense (SDCO), Odense, Denmark; 9 Department of Health and Caring Sciences, Western Norway University of Applied Sciences, Bergen, Norway; 10 Division of Endocrinology and Diabetology, Department of Internal Medicine, Medical University of Graz, Graz, Austria; 11 Department of Diabetes, St Vincent’s University Hospital, Dublin, Ireland

**Keywords:** Autonomic, Gender, Hypoglycaemia, Hypoglycaemia symptoms, Neuroglycopenic, Person-reported hypoglycaemia, Sensor-detected hypoglycaemia

## Abstract

**Aims/hypothesis:**

The aim of this study was to explore associations between gender and hypoglycaemia experience among people with type 1 diabetes or insulin-treated type 2 diabetes in a free-living environment in the Hypo-METRICS study.

**Methods:**

The Hypo-METRICS study was a 10-week prospective, cross-sectional observational study of the hypoglycaemia experience. Participants (274 with type 1 diabetes and 321 with type 2 diabetes) wore blinded continuous glucose monitor (CGM) devices and recorded their hypoglycaemia symptoms in real time using a bespoke Hypo-METRICS app. Symptomatic hypoglycaemia was defined as a glucose level <4 mmol/l or any episode of hypoglycaemic symptoms that was resolved by carbohydrate ingestion. Hypoglycaemia symptoms were defined as autonomic (hunger, sweating, shaking, palpitations) or neuroglycopenic (confusion, difficulty speaking, coordination difficulties, headache). Differences between genders were assessed using χ^2^, Fisher’s exact and Wilcoxon rank-sum tests and combination analyses.

**Results:**

In the type 1 diabetes cohort, 54% were women and 76% of the entire cohort used CGM routinely. In the type 2 diabetes cohort, 63% were men and 41% of the entire cohort used CGM routinely. There were no significant differences in time in hypoglycaemia between genders in either cohort (all *p*>0.05), but women reported more episodes of symptomatic hypoglycaemia per week (4.3 vs 3.3 episodes/week in the type 1 diabetes cohort [p=0.003]; 1.4 vs 1.0 episodes/week in the type 2 diabetes cohort [*p*=0.006]). In the type 1 diabetes cohort, women reported a broader range of symptom combinations, spanning autonomic and neuroglycopenic symptoms. Confusion was the only reported symptom of hypoglycaemia in 0.9% of episodes in women and 2.3% of episodes in men. In the type 2 diabetes cohort, there were more symptom combinations, and both genders reported a combination of autonomic and neuroglycopenic symptoms. Confusion alone accounted for 0.3% of symptomatic episodes in men in the type 2 diabetes cohort; confusion alone was not observed in women.

**Conclusions/interpretation:**

Despite similar exposures to hypoglycaemia, there were differences in hypoglycaemia symptoms between genders in this free-living environment. Further work is needed to elucidate the potential underlying causes of these findings.

**Graphical Abstract:**

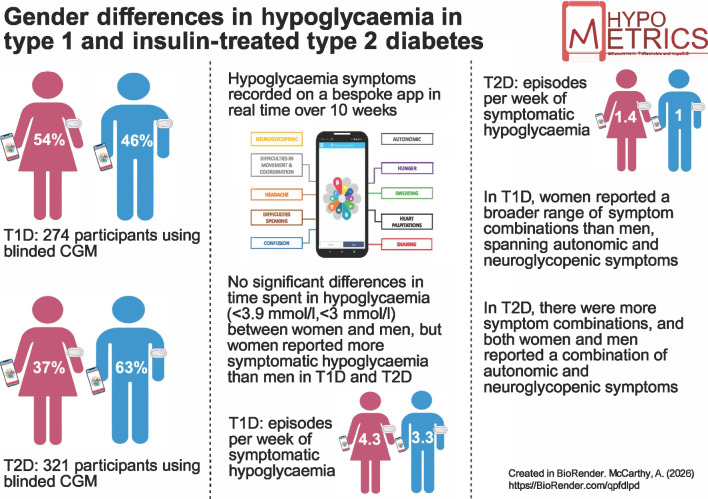

**Supplementary Information:**

The online version contains supplementary material available at 10.1007/s00125-026-06775-6.


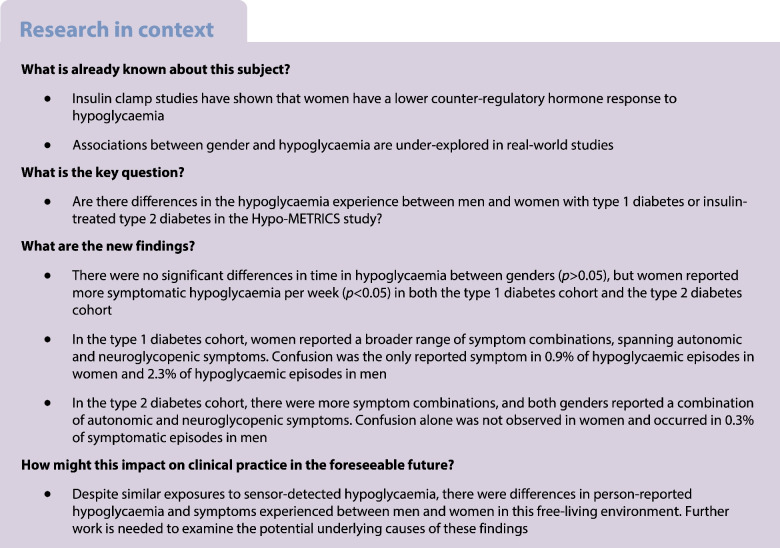


## Introduction

There is growing evidence suggesting that gender differences play a role in the management of chronic disease, including in diabetes [1]. Studies have suggested differences in the hypoglycaemia experience between the genders, with some studies suggesting a higher risk of severe hypoglycaemia in women compared with men [2] and higher rates of level 2 hypoglycaemia (<3 mmol/l) in self-reported data [3], but the results remain conflicting and scarce [4]. Potential differences in hypoglycaemia are important, as hypoglycaemia can compromise quality of life and diabetes management [5–7].

Current consensus guidelines classify hypoglycaemia into three levels: level 1, representing an alert value, defined as a glucose value of <3.9 mmol/l (<70 mg/dl); level 2, clinically important hypoglycaemia, defined as a glucose level of <3 mmol/l (<54 mg/dl); and level 3, severe hypoglycaemia, defined as cognitive impairment requiring external assistance for recovery [8, 9]. Sensor-detected hypoglycaemia (SDH) is defined as periods of >15 min below these thresholds on continuous glucose monitor (CGM) devices [9]. Using these definitions, there is a large discordance between SDH and person-reported hypoglycaemia (PRH) [10]. Additionally, individuals living with diabetes sometimes report hypoglycaemic symptoms at glucose levels >3.9 mmol/l [6, 11, 12]. While asymptomatic hypoglycaemia may have significant biological effects [13–15], the impact of hypoglycaemia on daily functioning is dependent on the symptomatic experience of hypoglycaemia [16].

Hypoglycaemia symptoms may be classified as either autonomic or neuroglycopenic. Autonomic signs and symptoms may be adrenergic, including shaking and palpitations, or cholinergic, including hunger and sweating [17]. Neuroglycopenia results from deprivation of glucose in the central nervous system (CNS), and its signs and symptoms include confusion, difficulty speaking and headache. These complications, particularly confusion, can impair behavioural defences [18] and lead to serious consequences if the person is unable to treat their hypoglycaemia [17]. As in the general population, these symptoms can also occur for other reasons in people living with diabetes, not related to hypoglycaemia [19].

Experimental studies in individuals with type 1 diabetes have shown differences in hypoglycaemic responses between the genders, with women showing a lower counter-regulatory hormone response to hypoglycaemia than men in insulin clamp studies [20, 21] but similar autonomic symptom responses [22]. While not fully understood, the mechanisms governing gender differences in hypoglycaemic response are thought to be related to reduced CNS drive and the effect of oestrogen on modulating the CNS response to hypoglycaemia, contributing to a weaker counter-regulatory response in women [23–25]. Data on gender-related differences in symptoms of hypoglycaemia in real-world studies using CGM are limited.

Using data from the Hypo-METRICS (Hypoglycaemia MEasurement, ThResholds and ImpaCtS) study [26], we aim to explore differences in the frequency and duration of SDH and PRH episodes, as well as differences in symptomatology, between women and men.

## Methods

### Study design

Hypo-METRICS was a 10-week multinational observational study conducted at nine locations across five European countries (Austria, Denmark, France, the Netherlands and the UK) from October 2020 to August 2022. The study protocol received approval from the ethics committees in each participating country. The trial is registered on ClinicalTrials.gov (NCT04304963). Written informed consent was obtained from all participants. Details of the protocol have been published previously [26].

### Study participants

Key inclusion criteria for the study were age 18–85 years, having type 1 diabetes or type 2 diabetes requiring at least one daily insulin injection, and having experienced at least one episode of hypoglycaemia (either measured or symptomatic) in the preceding 3 months. Key exclusion criteria included an eGFR <30 ml/min per 1.73 m^2^ and the use of automated insulin delivery systems. The complete list of inclusion and exclusion criteria has been published previously [26]. Our study population is broadly representative of the larger population of interest (people living with type 1 diabetes or insulin-treated type 2 diabetes).

### Study procedure

The protocol involved collection of demographic information, clinical data and blood samples for determination of HbA_1c_ and renal function. Participants wore a blinded study sensor (Freestyle Libre 2; Abbott, Alameda, CA, USA) for 10 weeks, and also continued using their usual glucose monitoring methods (capillary blood glucose [CBG] readings or personal CGM devices) for routine diabetes management. Participants used a bespoke Hypo-METRICS smartphone application [27] to record episodes and details of hypoglycaemia experienced by participants in real time or near real time. When participants reported a PRH episode on the Hypo-METRICS app, they were prompted to select and grade the intensity (from ‘not at all’ to ‘very much’) for any number of the following eight symptoms: hunger, sweating, shaking, palpitations (autonomic) and confusion, difficulty speaking, coordination difficulties, headache (neuroglycopenic). Details of app development and content validity have been published previously [27–29]. Gender and ethnicity were defined based on self-identification.

### Sensor-detected hypoglycaemia

Blinded study sensors collected CGM data at 5 min intervals. SDH episodes <3.9 and <3 mmol/l were defined according to the Advanced Technologies and Treatments for Diabetes (ATTD) consensus guidelines as a minimum of 15 min below the threshold, resolving when glucose levels exceeded the threshold for >15 min [9].

### Person-reported hypoglycaemia

PRH events were defined based on input from experts and the Hypo-RESOLVE patient advisory committee as symptomatic episodes resolved by carbohydrate ingestion, or measured glucose <3.9 mmol/l on routine glucose monitoring via clinical CGM or CBG device. The threshold was set at <3.9 mmol/l to align with clinical education programmes for diabetes management at the study locations. PRH episodes were further subdivided into symptomatic (detected on the basis of symptoms) or technology-detected (detected by alerts or chance checks of capillary or CGM glucose without symptoms).

### Data analysis

Participants with fewer than 14 days of CGM data were excluded from the analyses. The results are presented as median (IQR) and percentages unless otherwise specified. The χ^2^, Fisher’s exact and Wilcoxon rank-sum tests were used to test for differences in baseline characteristics between genders. χ^2^ tests with Bonferroni correction for multiple comparisons were used to assess for differences in individual symptoms based on gender. Conjunction analyses using UpSet plots created in R Studio were used for descriptive analysis of differences in the top 25 symptom combinations based on gender. Statistical tests were performed using R Studio [30].

## Results

In total, 602 participants (277 with type 1 diabetes and 325 with insulin-treated type 2 diabetes) took part in the 10 week study. Of these, five had fewer than 14 days of CGM data, and two did not identify as women or men, leaving 274 with type 1 diabetes and 321 with type 2 diabetes. In total, 37,386 days of CGM data were collected, comprising 17,117 days for the type 1 diabetes cohort and 20,269 days for the type 2 diabetes cohort. Symptom details were reported in 80% of PRH episodes. The baseline characteristics for the two cohorts are shown in Table 1.

**Table 1:** Baseline characteristics in individuals with type 1 diabetes and those with insulin-treated type 2 diabetes by gender

Characteristic	Type 1 diabetes			Insulin-treated type 2 diabetes		
	Women (*n*=148)	Men (*n*=126)	*p* value	Women (*n*=119)	Men (*n*=202)	*p* value
Age, years	44 (28–53)	50 (33–59)	0.017	60 (52–66)	64 (58–71)	0.001
Duration of diabetes, years	22 (10–34)	20 (9–37)	0.894	19 (12–26)	19 (13–24)	0.88
Impaired awareness of hypoglycaemia	20 (30)	21 (27)	0.93	29 (34)	26 (53)	0.745
Ethnicity			0.252			0.06
White	91 (134)	86 (108)		86 (102)	93 (187)	
Other^a^	9 (14)	14 (18)		14 (17)	7 (15)	
Country			0.015			0.34
UK	65 (96)	46 (58)		48 (57)	36 (72)	
The Netherlands	9 (14)	17 (21)		25 (30)	34 (68)	
Austria	11 (17)	12 (15)		15 (18)	17 (34)	
Denmark	7 (10)	16 (20)		11 (13)	12 (25)	
France	7 (11)	10 (12)		1 (1)	1 (3)	
Employment			0.228			0.009
Employed	71 (105)	67 (84)		42 (50)	32 (65)	
Retired	14 (21)	21 (26)		41 (49)	58 (117)	
Full-time education	7 (11)	3 (4)		1 (1)	2 (4)	
Unemployed	7 (11)	10 (12)		16 (19)	8 (16)	
Highest level of education achieved			0.923			0.87
Primary school	1 (1)	2 (2)		9 (11)	12 (25)	
Secondary school	22 (33)	25 (32)		31 (37)	32 (65)	
Undergraduate degree	45 (67)	43 (54)		38 (45)	33 (67)	
Postgraduate degree	26 (39)	25 (31)		12 (14)	11 (22)	
Other	5 (8)	6 (7)		10 (12)	11 (23)	
Glucose monitoring			0.002			0.92
CBG monitoring	17 (25)	33 (42)		59 (70)	59 (120)	
CGM	83 (123)	67 (84)		41 (49)	41 (82)	
Monitoring frequency						
CBG, per day	4.9 (3.5–6.3)	4 (3.1–6)	0.539	2.1 (1.4–3)	1.9 (1–3)	0.38
Flash glucose monitoring scans, per week	11 (8–16)	11 (8–16)	0.898	7 (5–10)	7 (5–10)	0.96
Insulin delivery			0.007			0.74
Multiple daily injections	57 (85)	73 (92)		97 (116)	97 (196)	
Insulin pump	43 (63)	27 (34)		3 (3)	3 (6)	
HbA_1c_			0.661			0.44
mmol/mol	57 (50–63)	57 (51–63)		56 (51–67)	60 (51–67)	
%	7.4 (6.7–7.9)	7.4 (6.8–7.9)		7.3 (6.8–8.3)	7.6 (6.8–8.3)	
Glycaemic indices						
% TIR (3.9–10 mmol/l)	60 (51–72)	60 (53–71)	0.58	76 (58–85)	65 (49–77)	<0.001
% TAR (>10 mmol/l)	33 (20–46)	31 (20–42)	0.46	22 (12–40)	32 (20–49)	<0.001
% Time in level 1 hypoglycaemia (<3.9 mmol/l)	4.2 (2.6–7.6)	4.5 (2.3–8.1)	0.68	1.6 (0.7–3.3)	1.3 (0.4–3.3)	0.18
% Time in level 2 hypoglycaemia (<3 mmol/l)	0.6 (0.20–1.30)	0.55 (0.13–1.58)	0.98	0.1 (0.00–0.30)	0.1 (0.00–0.30)	0.30
Beta-blocker usage			0.248			0.014
Yes	6 (9)	10 (13)		24 (29)	38 (77)	
No	89 (131)	81 (102)		68 (81)	54 (110)	
Missing data	5 (8)	9 (11)		8 (9)	7 (15)	

Values for continuous variables are median (IQR); values for categorical variables are presented as percentages, with the number of individuals given in parentheses

^a^‘Other’ included individuals of Asian and Black ethnicities and mixed ethnic backgrounds

### Type 1 diabetes cohort

In the type 1 diabetes population, women comprised 54% of the cohort and were younger than the men in that cohort (44 vs 50 years; *p*=0.017), but diabetes duration did not differ significantly between women and men (22 vs 20 years; *p=*0.894). More women used routine CGM (83% vs 67%; *p*=0.002) and insulin pumps (43% vs 27%; *p*=0.007). The median app completion rate was 90% (IQR 84–94%) in women and 90% (IQR 84–96%) in men (*p*=0.58). There were no statistically significant differences between the genders for time in range (TIR), time above range (TAR) or time below range (TBR <3.9 or <3 mmol/l) (Table 1).

**Fig. 1:**
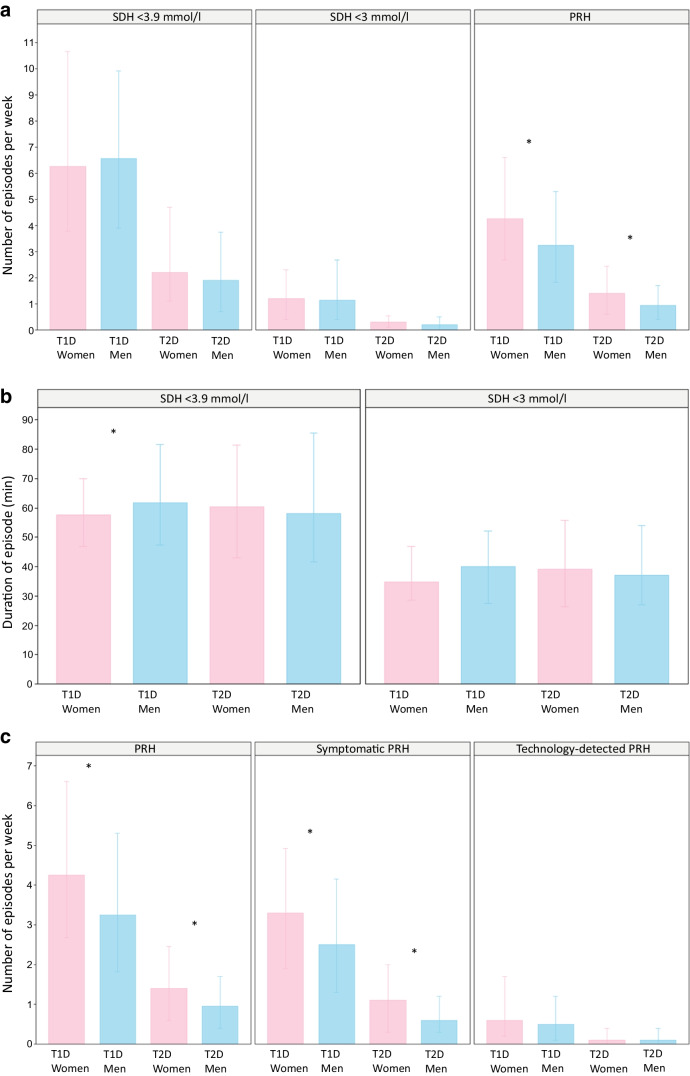
(**a**) Rates of SDH (<3.9 or <3 mmol/l) and PRH episodes in women and men with type 1 diabetes (T1D) or insulin-treated type 2 diabetes (T2D). (**b**) Duration of SDH (<3.9 or <3 mmol/l) in women and men with type 1 diabetes or insulin-treated type 2 diabetes; (**c**) Rates of subtypes of PRH in women and men with type 1 diabetes or insulin-treated type 2 diabetes. Values are medians and IQRs. *p<0.05

There were no significant differences between women and men for the median weekly rates of SDH episodes <3.9 mmol/l (6.2 episodes/week in women [IQR 3.8–10.7] vs 6.5 episodes/week in men [IQR 3.9–9.9]; *p*=0.76) or SDH episodes <3 mmol/l (1.2 episodes/week in women [IQR 0.4–2.3] vs 1.2 episodes/week in men [IQR 0.4–2.7]; *p*=0.77) (Fig. 1a). The median duration of SDH episodes <3.9 mmol/l was shorter for women at 58 min (IQR 47–70) compared with 62 min (IQR 47–82) in men (*p*=0.037). For SDH episodes <3 mmol/l, the median duration was 35 min (IQR 29–47) in women and 40 min (IQR 27–52) in men (*p*=0.36) (Fig. 1b). There were no differences in the proportion of SDH episodes <3.9 mmol/l (43% vs 38%; *p*=0.11) or SDH episodes <3 mmol/l (50% vs 44%; *p*=0.69) based on gender.

The median weekly rates of PRH episodes were higher in women than in men (4.3 episodes/week [IQR 2.7–6.6] in women vs 3.3 episodes/week [IQR 1.8–5.3] in men; *p*=0.003) as were the weekly rates of PRH episodes identified by symptoms only (3.3 episodes/week [IQR 1.9–4.9] in women vs 2.5 episodes/week [IQR 1.3–4.2] in men;* p*=0.005). There were no significant differences between the genders for rates of technology-detected PRH (0.6 episodes/week [IQR 0.2–1.7] in women vs 0.5 episodes/week [IQR 0.1–1.2] in men; *p*=0.11) (Fig. 1c). The differences in rates of hypoglycaemia while participants were awake or asleep are presented in electronic supplementary material (ESM) Table 1.

**Fig. 2:**
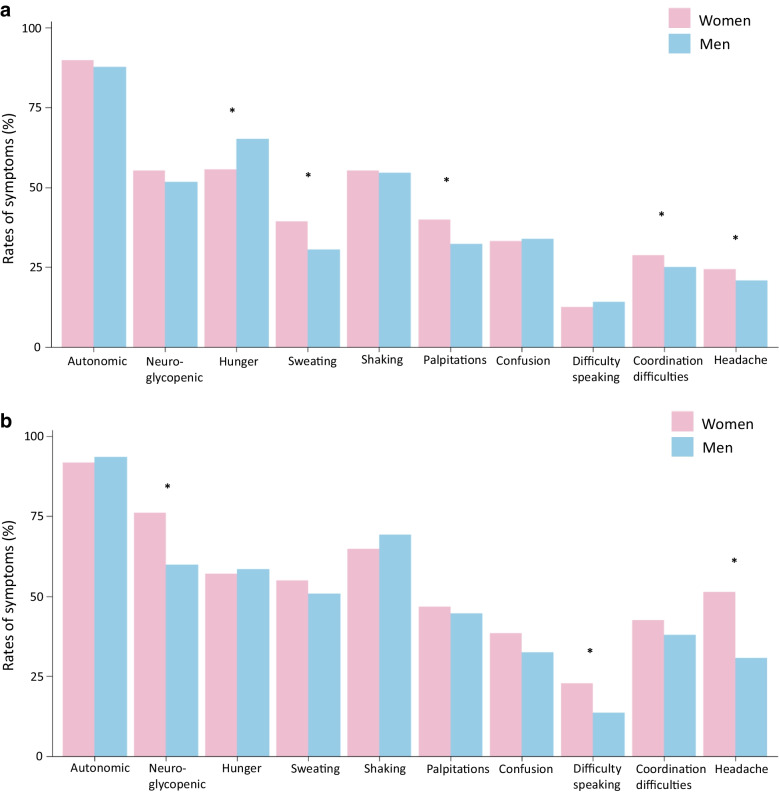
Differences in overall rates of autonomic and neuroglycopenic symptoms, and rates of each of the eight individual hypoglycaemia symptoms (hunger, sweating, shaking, palpitations, confusion, difficulty speaking, coordination difficulties and headache) based on gender in (**a**) individuals with type 1 diabetes and (**b**) individuals with insulin-treated type 2 diabetes. *p<0.05

Although there were no differences in overall rates of autonomic symptoms (89.9% vs 87.9%;* p*=0.32) or neuroglycopenic symptoms (55.2% vs 51.8%; *p*=0.17) based on gender after Bonferroni correction, there were differences in rates of individual autonomic and neuroglycopenic symptoms. Women reported higher rates of sweating (39.3% vs 30.6%; *p*<0.001), palpitations (39.5% vs 32.2%; *p*<0.001), coordination difficulties (28.7% vs 25%; *p*=0.041) and headache (24.3% vs 20.7%; *p*=0.034) compared with men. Men reported higher rates of hunger (55.7% vs 65%; *p*<0.001). There were no differences between the genders for shaking (*p*=0.621), confusion (*p*=0.652) or difficulty speaking (*p*=0.128) (Fig. 2a).

**Fig. 3:**
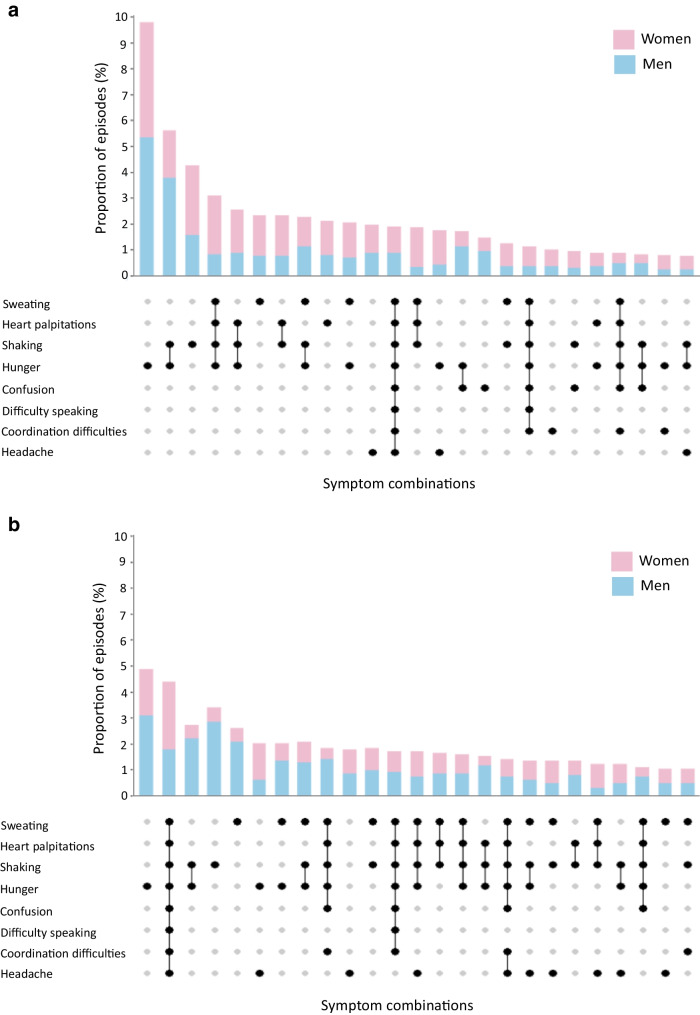
Descriptive analysis showing the frequency of the top 25 most common hypoglycaemia symptom combinations in (**a**) individuals with type 1 diabetes and (**b**) individuals with insulin-treated type 2 diabetes, with each stacked bar showing the percentages of women and men who have that combination of symptoms

The 25 most common combinations of symptoms are presented in Fig. 3a. Hunger as the only reported symptom was most common, accounting for 9.8% of all symptomatic hypoglycaemia episodes (7.6% of all combinations of symptoms reported by women, 12.9% of all combinations reported by men). Shaking and hunger was the next most common combination. The top 10 combinations of symptomatic hypoglycaemia included only autonomic symptoms. Confusion was the only reported symptom in 1.5% of all PRH episodes in the type 1 diabetes cohort (0.9% of women, 2.3% of men). The top five symptom combinations represented 25% of all PRH episodes.

### Insulin-treated type 2 diabetes cohort

In the type 2 diabetes population, men comprised 63% of the cohort and were older than the women (64 vs 60 years; *p*=0.001), but diabetes duration did not differ between the groups based on gender (both 19 years; *p*=0.88). The median app completion rate was 92% (IQR 86–96%) in women and 91% (IQR 84–96%) in men (*p*=0.83). The median TIR was higher in women (76% vs 65%; *p*<0.001) and the median TAR was higher in men (32% vs 22%; *p*<0.001). There were no statistically significant differences in TBR <3.9 mmol/l or TBR <3 mmol/l based on gender (Table 1).

There were no significant differences between women and men for the median weekly rates of SDH episodes <3.9 mmol/l (2.2 episodes/week [IQR 1.1–4.8] in women vs 1.9 episodes/week [IQR 0.7–3.8] in men; *p*=0.08) or SDH episodes <3 mmol/l (0.3 episodes/week [IQR 0.1–0.56] in women vs 0.2 episodes/week [IQR 0.0–0.51] in men; *p*=0.13) (Fig. 1a). There were no significant differences between the genders for the median duration of SDH episodes <3.9 mmol/l (60 min [IQR 43–81] in women vs 58 min [42–86] in men; *p*=0.7) or SDH episodes <3 mmol/l (39 min [IQR 26–56] in women vs 37 min [IQR 27–54] in men; *p*=0.33) (Fig. 1b). There were no differences in the proportion of SDH episodes <3.9 mmol/l (24 vs 22%; *p*=0.42) or SDH episodes <3 mmol/l (14 vs 26%; *p*=0.28) based on gender.

The median weekly rates of PRH episodes were higher in women than in men (1.4 episodes/week [IQR 0.6–2.5] in women vs 1.0 episode/week [IQR 0.4–1.7] in men; *p*=0.006) as were the weekly rates of PRH episodes identified by symptoms only (1.1 episodes/week [IQR 0.3–2.0] in women vs 0.6 episodes/week [IQR 0.3–1.2] in men; *p*=0.003). There were no significant differences between the genders for rates of technology-detected PRH (0.1 episodes/week [IQR 0–0.4] in both women and men; *p*=0.79) (Fig. 1c). The differences in rates of hypoglycaemia while participants were awake and asleep are presented in ESM Table 1.

Women reported higher rates of neuroglycopenic symptoms (76.1% vs 59.8%; *p*<0.001) but not autonomic symptoms (91.8% vs 93.7%; *p*=0.156) compared with men after Bonferroni correction. Women had higher rates of difficulty speaking (22.75% vs 13.5%; *p*<0.001) and headache (51.4% vs 30.7%; *p*<0.001). There were no significant differences in sweating (55% vs 50.8%; *p*=0.1), palpitations (46.75% vs 44.6%; *p*=0.42), shaking (64.4% vs 69.3%; *p*=0.65), hunger (57.2% vs 58.5%; *p*=0.621), coordination difficulties (42.5% vs 37.97%; *p*=0.69) or confusion (38.5% vs 32.5%; *p*=0.13) between the groups based on gender (Fig. 2b).

The most common 25 symptom combinations are presented in Fig. 3b. Hunger as the only reported symptom was the most common, accounting for 4.9% of all PRH episodes (3.6% of all combinations of symptoms reported by women, 6.1% of all combinations reported by men). The combination of all eight symptoms was the next most common combination. Neuroglycopenic symptoms featured more frequently in combination with other symptoms in the type 2 diabetes cohort compared with the type 1 diabetes cohort, and were present in four of the top 10 combinations.

## Discussion

In this analysis, we demonstrate that, despite similar rates of hypoglycaemia overall between the genders, women reported more symptomatic hypoglycaemia and a different symptom profile compared with men.

In both the type 1 diabetes cohort and the type 2 diabetes cohort, women had similar hypoglycaemia exposure to men, with no significant differences in rates of SDH episodes <3.9 mmol/l, SDH episodes <3 mmol/l, TBR <3.9 mmol/l or TBR <3 mmol/l. Existing data on the differences in hypoglycaemia rates and experience between the genders are limited. In the type 1 diabetes cohort, a retrospective analysis based on self-reported data demonstrated that women reported more level 2 hypoglycaemia (<3 mmol/l) than men, in a population where 87% were using routine CGM [3]. There is also evidence from a study in individuals with type 1 diabetes using CGM where participants were assigned to one of three exercise programmes (aerobic, high-intensity interval or resistance training) that men were less likely to meet the ADA/EASD targets for time in level 1 hypoglycaemia (SDH <3.9 mmol/l) [31]. For type 2 diabetes, there are no CGM data on hypoglycaemia and gender differences, although there are data that show that incidence rates of severe hypoglycaemia and severe nocturnal hypoglycaemia are higher in women [32]. Our findings add to this limited literature, and align with the results of insulin clamp studies showing that women have a lower counter-regulatory hormone response to hypoglycaemia than men [20, 21] but similar autonomic symptom responses [22].

While women had similar hypoglycaemia exposure to men in our study population, women in both the type 1 diabetes cohort and the type 2 diabetes cohort experienced more symptomatic hypoglycaemia. There are several potentially explanatory factors, including differences in interoceptive awareness [33] and health literacy [34], and the impact of menstruation and menopause in women [35, 36]. Interoceptive awareness is a person’s perception of signals originating from within the body [33] and is important in the identification of hypoglycaemia [37]. There is evidence to suggest that women have greater interoceptive awareness than men, and are more likely to engage in actions to detect possible symptoms of diseases and to interpret sensations as a warning [38]. Women often have better health literacy than men [34] and have higher rates of attendance at structured diabetes education programmes such as the dose adjustment for normal eating programme (DAFNE) [39], which may contribute to a greater knowledge of hypoglycaemia symptoms, as well as the potential for reporting bias. In our type 1 diabetes cohort, although women reported more symptomatic hypoglycaemia, they spent the same amount of time in hypoglycaemia as men. Given women’s higher levels of interoception and health literacy, they may be more likely than men to look out for symptoms of hypoglycaemia. As there was no difference in the number of technology-detected PRH episodes between women and men, despite higher rates of clinical CGM use in women in the type 1 diabetes cohort, it is possible that CGM is not the reason for the difference in hypoglycaemia experience between women and men, but rather that women may be more likely to get a clinical CGM because they report more symptomatic hypoglycaemia.

There is limited evidence on the impact of the menstrual cycle and menopause on hypoglycaemia symptoms in women. In women without diabetes, research suggests that the counter-regulatory hormonal response to hypoglycaemia is similar in the follicular and luteal phases of the menstrual cycle [40]. However, hormonal fluctuations during the menstrual cycle can affect blood glucose levels and insulin sensitivity [41], and oestrogen has a modulating effect on the CNS response to hypoglycaemia, contributing to a weaker counter-regulatory response in women [23–25]. There is evidence that the differences in counter-regulatory hormone response and sympathetic nervous system (SNS) activation seen between women and men are not observed between postmenopausal women and men [42], suggesting that gender differences in the hypoglycaemia experience may be less pronounced with advancing age. Given the age profiles of our participants, the majority of the women with type 1 diabetes were probably premenopausal, while the majority of women with type 2 diabetes were probably postmenopausal, although we do not have data on the menopausal status of participants. We can speculate that this may have impacted their symptoms, but the impact of the menstrual cycle and menopause on hypoglycaemia symptoms has not been tested in free-living conditions, and this represents a gap in the literature.

In the type 1 diabetes cohort, our analysis demonstrates that women have higher rates of certain autonomic symptoms than men, including sweating and palpitations. Insulin clamp studies have demonstrated that women have decreased SNS activation with lower levels of adrenaline (epinephrine) in response to hypoglycaemia, but increased sensitivity to the SNS drive for autonomic symptom awareness during hypoglycaemia, such that autonomic symptom responses are similar between the genders [22]. In the type 1 diabetes cohort, women were more likely to report a broader range of hypoglycaemia symptom combinations, spanning both autonomic and neuroglycopenic symptoms, while men had a narrower breadth of symptom combinations. The wider variety of hypoglycaemia symptoms in women may explain why women report more symptomatic hypoglycaemia, as they link more symptoms to the experience of hypoglycaemia. The top 10 combinations of symptoms in both women and men with type 1 diabetes all comprise autonomic symptoms. These symptoms may arise from sympathoadrenal involvement in response to perceived hypoglycaemia [17]. In the type 2 diabetes cohort, there was much more heterogeneity in the symptom combinations than in the type 1 diabetes cohort. Women and men both reported a combination of autonomic and neuroglycopenic symptoms, and neuroglycopenic symptoms occurred earlier in the top symptom combinations in the type 1 diabetes cohort than in the type 2 diabetes cohort.

In the type 1 diabetes cohort, women had higher rates of coordination difficulties and headache compared with men, whereas in the type 2 diabetes cohort, women had higher overall rates of neuroglycopenic symptoms, including higher rates of headache and difficulty speaking during hypoglycaemia. Neuroglycopenia refers to CNS deprivation of glucose, and complications from cortical neuroglycopenia, particularly confusion, can impair behavioural defences [18]. Insulin clamp studies demonstrate that oestrogen appears to be a major mediator of the reduced neuroendocrine and metabolic responses to hypoglycaemia in women, contributing to a weaker counter-regulatory response in women [23–25]. Evidence regarding differences in neuroglycopenic symptoms between the genders is limited, but the evidence that exists does not report a difference in neuroglycopenic symptoms between the genders [43]. While overall rates of neuroglycopenic symptoms were higher in women, neuroglycopenic symptoms occurring in isolation were rare for both women and men, including isolated confusion. Confusion is a concerning neuroglycopenic symptom, as it can lead to serious neurological consequences if the person is unable to treat their hypoglycaemia [18]. It is particularly concerning if it occurs alone, as without the ‘warning’ from other autonomic or neuroglycopenic symptoms, the person may be unaware of and unable to treat their hypoglycaemia. In the type 1 diabetes cohort, confusion was the only symptom in 1.5% of all PRH episodes, with a higher percentage in men (2.3%) compared with women (0.9%). This low percentage of confusion as the only symptom of hypoglycaemia is reassuring for clinical practice.

In the type 2 diabetes cohort, there was more heterogeneity in symptom combinations than observed in the type 1 diabetes cohort, as type 2 diabetes is a more heterogeneous condition [44]. There was also less of a difference observed between women and men. This may be partly attributable to the fact that perceived hypoglycaemia symptoms do not differ between genders in postmenopausal women compared with men [42], and that there is a balancing out of the differences in SNS activation. Neuroglycopenic symptoms occurred earlier in the top symptom combinations for both women and men than in the type 1 diabetes cohort. Confusion was the only symptom in 0.3% of all PRH episodes in the type 2 diabetes cohort (0% in women, 0.3% in men). The earlier neuroglycopenic symptoms in the type 2 diabetes cohort may be because these patients had lower rates of hypoglycaemia by all definitions than in the type 1 diabetes cohort, and were not as accustomed to experiencing hypoglycaemia; as such, they may have experienced the symptoms of hypoglycaemia more acutely and severely than in type 1 diabetes [45]. There is also evidence to suggest that the counter-regulatory deficiencies and reduced symptom responses that are common in individuals with type 1 diabetes are not present in people with long-standing insulin-treated type 2 diabetes [45]. The preserved adrenaline response in individuals with type 2 diabetes compared with those with type 1 diabetes may also be protective against confusion. The differences may also be important, as the individuals with type 2 diabetes are older, and counter-regulatory hormone and symptom responses to hypoglycaemia have been observed to wane over time [45]. Men with type 2 diabetes also had higher rates of beta-blocker usage, which may have blunted autonomic warning symptoms and affected symptom profiles. However, data from insulin clamp studies suggest that beta-blocker usage does not alter the ability to identify symptomatic hypoglycaemia [46].

It is important to recognise that the accuracy of CGM is reduced during hypoglycaemia, and there are reports of compression artefacts [47]. However, we would not expect differences in compression artefacts based on gender. The fact that episodes of SDH <2.2 mmol/l accounted for 302/35,710 SDH events, or 0.8% of our data [10], indicates the validity of our findings, even if a small number of these events were artefactual. Importantly, the Freestyle Libre sensor is the most widely used sensor in Europe, so our findings are relevant to the lived experience of all individuals who use this system.

The strengths of this analysis include the fact that it was based on a large multicentre multinational study. Both men and women were well-represented within the study, and detailed demographic information was captured, which allowed a robust descriptive analysis of this population. These data are unique as they capture the patient experience of hypoglycaemia in real time using ecological momentary assessments via the bespoke Hypo-METRICS app. This real-time description of symptom subtypes improves the accuracy of reporting, which would be difficult to capture in other study designs. The incorporation of both SDH and PRH data allows us to compare the objective and perceived experience of hypoglycaemia. The use of blinded CGM and the Hypo-METRICS app for real-time symptom monitoring reduces recall bias, and the high level of app engagement in this study demonstrates the usability of this app.

The limitations of this study include the fact that it is a post hoc analysis, and as such the findings presented should be considered exploratory rather than definitive. The study population is predominantly White European, and these findings may not apply to other regions and ethnicities. This study was also conducted during the COVID-19 pandemic, which led to significant changes in daily life and glycaemic outcomes, particularly in older age groups [48]. Because of technical constraints of the app, participants were only asked about eight common symptoms of hypoglycaemia, which may not fully reflect the myriad of symptoms experienced by people with diabetes. Nevertheless, the selection of these symptoms was informed by a literature review and the Hypo-RESOLVE patient advisory committee convened for the Hypo-METRICS study.

In conclusion, the most clinically relevant finding is that, for the same TBR, we may expect women to have a greater number of symptomatic episodes of hypoglycaemia with a much higher impact on their quality of life, and this should be taken into consideration during clinical care. Despite women reporting more symptoms, the majority of level 2 hypoglycaemia (SDH <3 mmol/l) is still asymptomatic, and this is an ongoing concern. The fact that confusion was rarely the only reported symptom is reassuring. More research is needed, particularly in the area of hypoglycaemia and women’s health, given the research gaps regarding the impact of the menstrual cycle and menopause on women’s experience of hypoglycaemia symptoms.

## Supplementary Information

Below is the link to the electronic peer-reviewed but unedited supplementary material.

ESM Table (PDF 91 KB)

## Data Availability

Data are accessible by application to the Hypo-RESOLVE data access committee (Pratik Choudhary [pratik.choudhary@leicester.ac.uk] or Bastiaan de Galen [Bastiaan.deGalan@radboudumc.nl]). Restrictions apply to the use of these data in accordance with participant consent, privacy regulations and data-sharing agreements.

## Abbreviations

CBG: Capillary blood glucose
CGM: Continuous glucose monitor(ing)
CNS: Central nervous system
PRH: Person-reported hypoglycaemia
SDH: Sensor-detected hypoglycaemia
SNS: Sympathetic nervous system
TAR: Time above range
TBR: Time below range
TIR: Time in range
