# Supplementary material for: Gender differences in hypoglycaemia in type 1 and insulin-treated type 2 diabetes: the Hypo-METRICS study
Source: Diabetologia. 2026 Jun 20;69(9):2445–57. doi: 10.1007/s00125-026-06775-6 (PMC13424728; doi:10.1007/s00125-026-06775-6)
Supplement: Supplementary file 1 — ESM Table (PDF 91 KB) [file 125_2026_6775_MOESM1_ESM.pdf]

Electronic Supplementary Material (ESM)

ESM Table 1: Differences in rates of hypoglycaemia based on gender while participants were awake and asleep

Type 1 diabetes			
**Hypoglycaemia metric**	**Women (*n*=148)**	**Men (*n* =126)**	***p* value**
Rate of SDH 3.9 mmol/l per week (awake)	3.7 (2.1-6.5)	3.6 (2.1-6.3)	0.907
Rate of SDH 3.9 mmol/l per week (asleep)	1.8 (0.7-2.9)	1.5 (0.8-2.6)	0.256
Rate of SDH 3.0 mmol/l per week (awake)	0.5 (0.1-1.1)	0.4 (0.1-1.3)	0.826
Rate of SDH 3.0 mmol/l per week (awake)	0.5 (0.1-0.9)	0.3 (0.1-0.9)	0.494
PRH (awake)	2.8 (1.6-4.9)	2.3 (1.2-3.9)	0.019
PRH (asleep)	0.75 (0.3-1.3)	0.5 (0.2-0.9)	0.005
**Insulin-treated type 2 diabetes**			
**Hypoglycaemia metric**	**Women (*n* =119)**	**Men (*n* =202)**	***p* value**
Rate of SDH 3.9 mmol/l per week (awake)	1 (0.3-2.2)	7 (0.1-1.9)	0.137
Rate of SDH 3.9 mmol/l per week (asleep)	0.8 (0.3-1.8)	0.6 (0.1-1.6)	0.083
Rate of SDH 3.0 mmol/l per week (awake)	0.1 (0-2)	0 (0-0.2)	0.257
Rate of SDH 3.0 mmol/l per week (awake)	0.1 (0-0.3)	0.1 (0-0.3)	0.13
PRH (awake)	1 (0.3-1.7)	0.5 (0.2-1.1)	0.002
PRH (asleep)	0.2 (0-0.4)	0.1 (0-0.4)	0.436
